# Shear Wave Elastography May Add a New Dimension to Ultrasound Evaluation of Thyroid Nodules: Case Series with Comparative Evaluation

**DOI:** 10.1155/2012/657147

**Published:** 2012-05-17

**Authors:** Rafal Z. Slapa, Antoni Piwowonski, Wieslaw S. Jakubowski, Jacek Bierca, Kazimierz T. Szopinski, Jadwiga Slowinska-Srzednicka, Bartosz Migda, R. Krzysztof Mlosek

**Affiliations:** ^1^Department of Diagnostic Imaging, Second Faculty of Medicine, Medical University of Warsaw, ul. Kondratowicza 8, 03-242 Warsaw, Poland; ^2^NZ0Z Almed, 37-500 Jarosław, Poland; ^3^Surgery Department, Solec Hospital, 00-382 Warsaw, Poland; ^4^Department of Dental and Maxillofacial Radiology, Institute of Stomatology, First Faculty of Medicine, Medical University of Warsaw, 02-006 Warsaw, Poland; ^5^Department of Endocrinology, Centre for Postgraduate Medical Education, 01-809 Warsaw, Poland

## Abstract

Although elastography can enhance the differential diagnosis of thyroid nodules, its diagnostic performance is not ideal at present. Further improvements in the technique and creation of robust diagnostic criteria are necessary. The purpose of this study was to compare the usefulness of strain elastography and a new generation of elasticity imaging called supersonic shear wave elastography (SSWE) in differential evaluation of thyroid nodules. Six thyroid nodules in 4 patients were studied. SSWE yielded 1 true-positive and 5 true-negative results. Strain elastography yielded 5 false-positive results and 1 false-negative result. A novel finding appreciated with SSWE, were punctate foci of increased stiffness corresponding to microcalcifications in 4 nodules, some not visible on B-mode ultrasound, as opposed to soft, colloid-inspissated areas visible on B-mode ultrasound in 2 nodules. This preliminary paper indicates that SSWE may outperform strain elastography in differentiation of thyroid nodules with regard to their stiffness. SSWE showed the possibility of differentiation of high echogenic foci into microcalcifications and inspissated colloid, adding a new dimension to thyroid elastography. Further multicenter large-scale studies of thyroid nodules evaluating different elastographic methods are warranted.

## 1. Introduction

The main reasons for the widespread use of thyroid sonography are availability, low cost, limited discomfort to the patient, and absence of ionizing radiation. Sonography has many favourable features, such as detection of nonpalpable nodules, estimation of nodule size/goiter volume, and guidance for fine needle biopsy (FNB). High-resolution ultrasound is very sensitive in detection of thyroid nodules, enabling differentiation of solid and liquid lesions. Consequently, the interobserver agreement is high [1].

However, with introduction of sonography it became evident that thyroid nodules are very common, with prevalence ranging from 17% to as much as 67% in some cohorts. Nodular goiter does not include thyroid cancer, but one of the main aims of the clinical evaluation is to exclude the risk of overlooking thyroid cancer which is much less prevalent than benign nodules. A hard thyroid nodule on neck palpation is suggestive of thyroid carcinoma [1].

Sonographic characteristics such as hypoechogenicity, microcalcifications, and increased nodular flow visualized by Doppler are all to some extent predictive of malignancy [1]. Microcalcifications visible on ultrasound examination are considered to be a specific feature of thyroid cancer (85,8–95%); however, the sensitivity of this sign is relatively low (29–59%) [2]. Presence of calcifications doubles the risk of malignancy, whereas microcalcifications increase the risk of thyroid cancer three-fold [3]. However, in pathologic examinations of benign thyroid nodules-inspissated colloid, fibrosis, and microcalcifications often coexist [4].

The estimation of tissue hardness is a very ancient diagnostic tool in medicine. Palpation—the earliest and most common form of tissue hardness estimation—was practiced by Egyptian physicians as early as 2600 BC [5].

A more recent and sophisticated method of imaging of tissue hardness is the technique known as elastography. The term “elastography” was coined by Ophir et al. [6] to refer to an ultrasound-based imaging technique, where local axial strains were estimated by computing the gradient of axial shifts in echo arrival times along the ultrasound beam direction following quasistatic tissue deformation. Elastography, however, has now been used as a more general term to identify methods that image tissue stiffness, using different imaging modalities for example, ultrasound, magnetic resonance imaging, optical coherence tomography, different perturbation techniques to deform tissue, based on the elasticity parameter being measured or imaged [7]. Roughly 20 years have elapsed since the first images depicting the local elastic properties of tissues were obtained. The first decade of development produced a remarkable proliferation of techniques and optimization strategies. In the second decade this trend continued, but with an important extension to dedicated platforms for conducting clinical trials in the hands of radiologists and skilled clinicians [8].

There are 3 main types of ultrasound elasticity imaging: elastography that tracks tissue movement during compression to obtain an estimate of strain (quasi-static elastography), sonoelastography that uses color Doppler to generate an image of tissue movement in response to external vibrations (harmonic elastography), and a technique tracking shear wave propagation through tissue to obtain the elastic modulus (transient elastography) [5].

The first commercially available, clinical ultrasound scanners with option of tissue elasticity evaluation were equipped with strain elastography. Application of strain elastography to thyroid nodules examination resulted in different results of evaluation of usefulness of this technique in regard to differential diagnosis of benign and malignant thyroid nodules [9–14]. Diagnostic value of strain elastography is limited in evaluation of small nodules, large nodules (with diameter approaching or exceeding the length of the probe), nodules with calcifications, and nodules with liquid content. Different results are obtained in different anatomical planes (e.g., axial versus sagittal), and the reproducibility is poor. Finally, multinodular goiters with scarce or no normal thyroid tissue for reference are difficult to evaluate [9, 11–16].

The new generation of elasticity imaging called supersonic shear wave elastography (SSWE) has been introduced since 2006 to imaging of superficial organs as breast and thyroid with high-frequency linear probes [17–21]. This type of transient elastography does not require the compression of the tissues during their elasticity examination. The obtained information is based on calculated elastic modulus (described in kPa) of the examined tissues. Based on multinational large-scale studies in the field of breast cancer detection and characterization, SSWE proved to be highly reproducible, and it increased specificity without loss of sensitivity [20, 21]. In the field of thyroid SSWE, it was proved that autoimmune thyroiditis does not hinder the evaluation of elasticity of thyroid nodules [20].

The aim of this study was to compare the usefulness of the new supersonic shear wave imaging elastography with strain elastography in evaluation of thyroid nodules.

## 2. Methods

During a few weeks trial time in 2010, four consecutive patients with single thyroid nodule (*n* = 1) and nodular goiter (*n* = 3) were evaluated. Approval for this study was obtained from the Ethics Committee of the Medical University of Warsaw, and all patients provided informed consent. The B mode and power Doppler ultrasound of whole thyroid and neck lymph nodes was performed. Six dominant thyroid nodules (in regard to B-mode and power Doppler ultrasound features) were evaluated with shear wave and strain elastography qualitatively and quantitatively as well as some with contrast-enhanced ultrasound (Sonovue (Bracco)). The examinations were performed with following scanner: Aixplorer (Supersonic Imagine Inc. France)—SSWE, Aplio XG (Toshiba, Japan)—strain elastography, Technos (Esaote, Italy)—contrast-enhanced ultrasound, with linear high-resolution transducers: 15–4 MHz, 18–7 MHz, and, 8–3 MHz respectively. For strain elastography, we adopted qualitative scale of Rubaltelli et al. with threshold score of 2/3 [22] and quantitative scale of Cantisani et al. with threshold thyroid tissue/nodule strain ratio of 2 [23] measured with Elasto-Q (Toshiba). For shear wave elastography, we adopted quantitative scale of Sebag et al. with the threshold stiffness (mean elastic modulus) of thyroid nodule of 65 kPa [19]. The final diagnosis was based on clinical evaluation, multiple FNB, 1 year followup, or surgery.

## 3. Results

**Figure 1:**
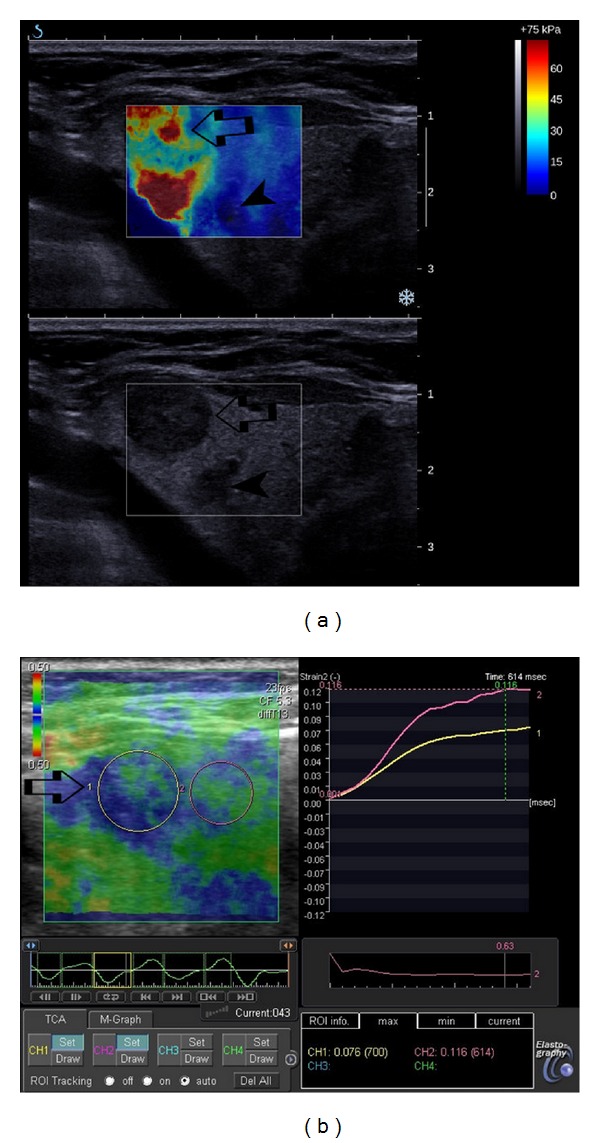
Diffuse sclerosing papillary carcinoma of the left lobe of the thyroid gland (arrow) in patient with multinodular goiter and multiple colloid nodules (arrow head). (a) on B-mode ultrasound (lower image), the cancer is less suspicious than some of the colloid nodules; however, on supersonic shear wave elastography (upper image) the nodule presents areas of high stiffness (over 75 kPa) indicating malignant lesion. The colloid nodule presents as a lesion with low stiffness. (b) on strain elastography, the carcinoma qualitatively and quantitatively presents features of soft lesion falsely indicating benign nature of the lesion.

**Figure 2:**
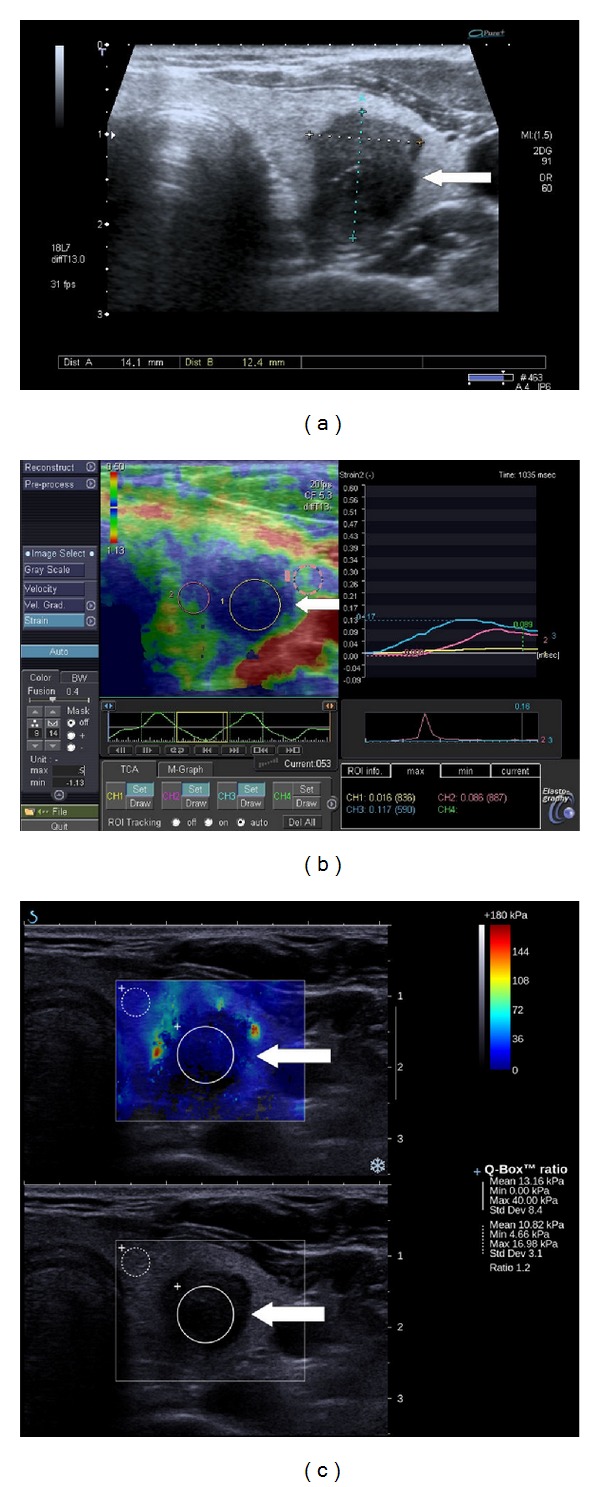
A benign nodule (arrow) of the left lobe of the thyroid gland (with massive degeneration demonstrated on contrast-enhanced ultrasound and in pathologic examination) in patient with multinodular goiter. (a) on B-mode axial section, the nodule is markedly hypoechoic, taller than wide, with somewhat ill-defined margins and high echogenic foci suggesting microcalcifications, a suspicious nodule. (b) on strain elastography, the nodule both qualitatively and quantitatively presents as very hard suspicious of cancer. (c) on supersonic shear wave elastography (upper image), the lesion is very soft, truly indicating its benign nature, with some stiff calcifications in the capsule.

Final diagnosis (pathology examination after surgery in 5 nodules, double FNB, and 1 year followup in 1 nodule) was established: 1 papillary carcinoma (Figure 1), 4 colloid nodules, and 1 benign nodule (Figure 2).

Shear weave elastography revealed 1 true positive and 5 true negative diagnoses in regard of thyroid cancer.

Strain elastography revealed 5 false positive and 1 false negative diagnoses.

**Figure 3:**
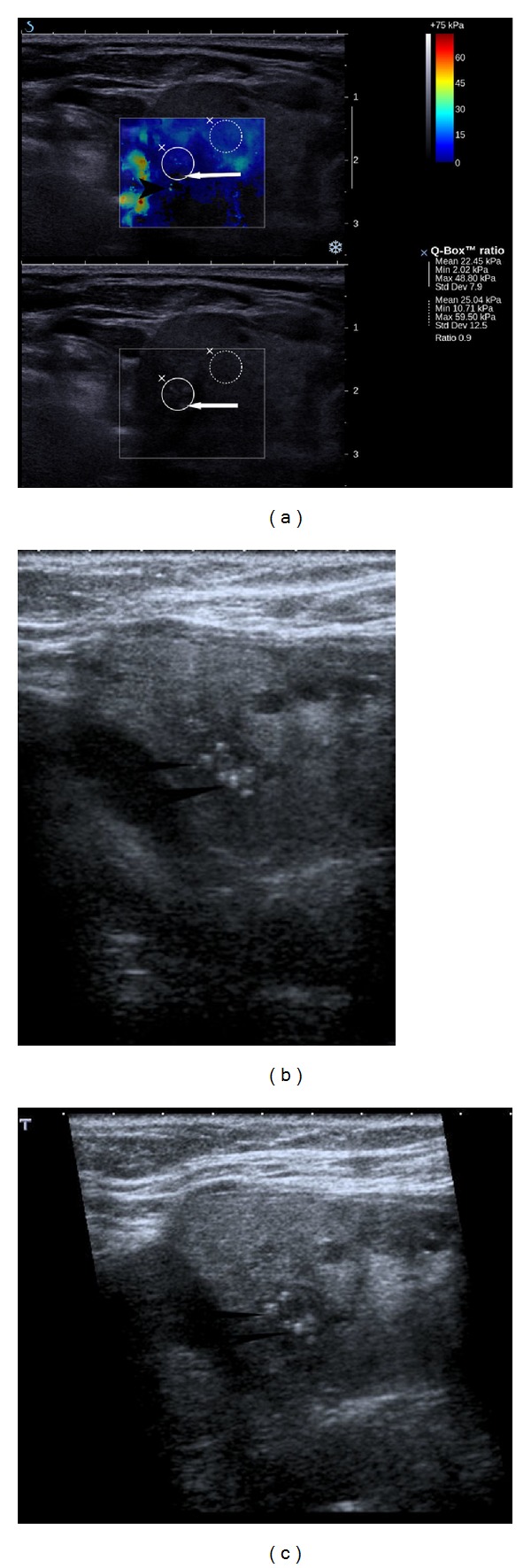
A colloid nodule in a patient with nodular goiter and papillary carcinoma. (a) on supersonic shear wave elastography (upper image) the colloid nodule (full circle) is very soft with punctuate increased stiffness (arrow head) representing microcalcifications (some not visible on B-mode lower image), in contrast to bigger high echogenicity foci (arrow), which are soft and represent inspissated colloid as evident on subsequent images. (b) presents comet tail artifacts (arrow heads) that change the direction with steering of ultrasound beam (c) indicating the nature of this artifact that is attributable to inspissated colloid in thyroid nodule.

False positive diagnoses with strain elastography were found in nodules with liquid (not evident on B-mode ultrasound) or degenerative content of the nodules visible on contrast-enhanced ultrasound and/or pathology examination. A novel finding were the punctate increased stiffness foci in microcalcifications seen in 4 nodules, some not visible on B-mode ultrasound as opposed to soft inspissated colloid foci visible on B-mode ultrasound in 2 nodules (Figure 3).

## 4. Discussion

Supersonic shear weave elastography consists of the generation of remote radiation force by focused ultrasonic beams, the so-called “pushing beams,” a patented technology called “Sonic Touch” [19]. Using Sonic Touch, ultrasound beams are successively focused at different depth in tissues. The source is moved at a speed that is higher than the speed of the shear waves that are generated. In this supersonic regime, shear waves are coherently summed in a “Mach cone” shape, which increases their amplitude and improves their propagation distance. For a fixed acoustic power at a given location, Sonic Touch increases shear wave generation efficiency by a factor of 4 to 8 compared to a nonsupersonic source [24]. After generation of this shear wave, an ultrafast echographic imaging sequence is performed to acquire successive raw radiofrequency dots at a very high-frame rate (up to 20000 frames per second). Based on Young's modulus formula, the assessment of tissue elasticity can be derived from shear wave propagation speed. A color-coded image is displayed, which shows softer tissue in blue and stiffer tissue in red. Quantitative information is delivered; elasticity is expressed in kilo-Pascal (kPa) [19].

This preliminary paper based on small number of cases indicates that SSWE indicated correctly thyroid nodules suspicious for cancer in contrast to strain elastography. False positives on strain elastography could be due to liquid or degenerative content of nodules.

**Figure 4:**
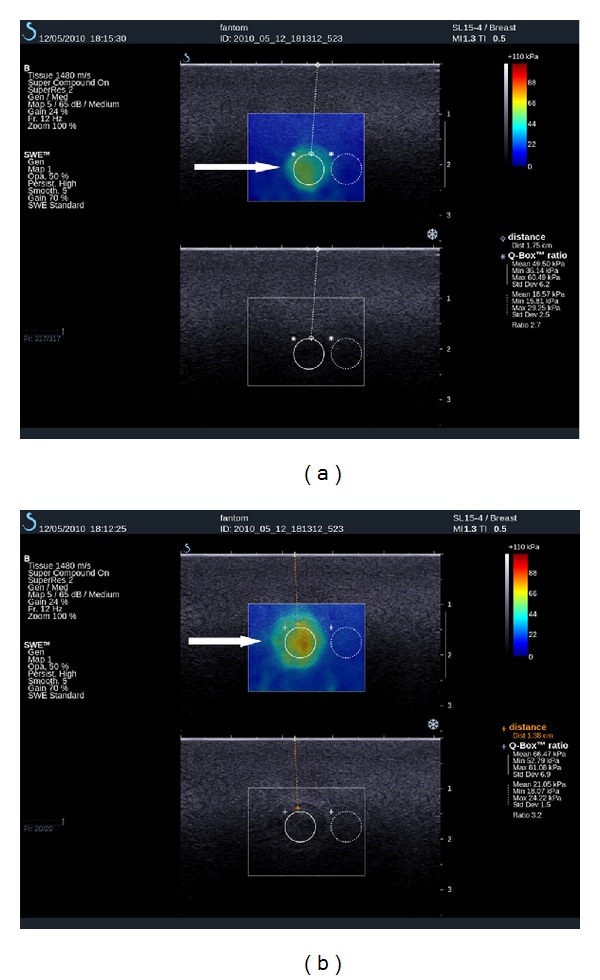
Phantom studies illustrating well-known physiologic phenomenon of increase of perceived tissue stiffness with applied increased pressure (e.g., during palpation of tissues). (a) on a breast phantom study a stiff inclusion (arrow) is seen with no pressure applied at ultrasound probe. The distance from the surface, the stiffness of the inclusion and surrounding medium (dotted circle) can be measured. (b) with application of pressure on the phantom with ultrasound probe, which is evident as a reduced distance of the inclusion to the surface of the phantom, the stiffness of the inclusion and surrounding medium is increased. Thus, the measurements of stiffness of tissues with supersonic shear wave elastography should be performed in a resting state without pressure on the ultrasound probe to avoid the unpredictable influence of the compression on the stiffness of the tissues.

**Figure 5:**
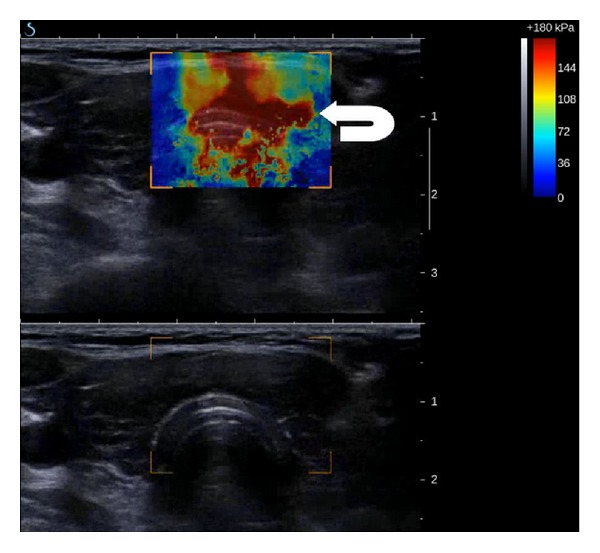
An artifact (arrow) from tissue compression on elastogram (upper image) in patient with Hashimoto thyroiditis, with no real focal lesions evident on B-mode ultrasound (lower image). A hard stiff pseudolesion is generated (see also Supplementary material 1 available online on doi: 10.1155/2012/657147). To avoid such artifacts no pressure on the probe during elasticity evaluation should be applied.

**Figure 6:**
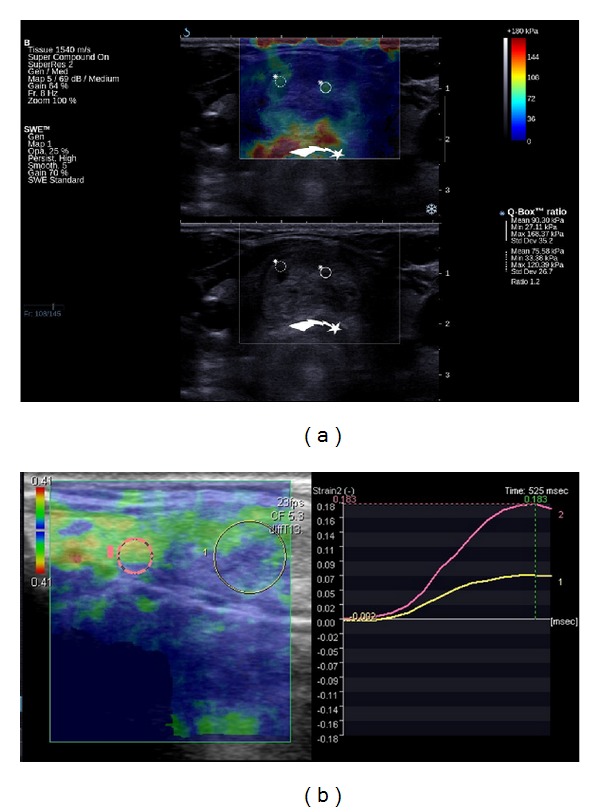
A benign thyroid nodule in a patient with solitary nodule in the thyroid isthmus that was suspicious on power Doppler ultrasound (not shown). The SSWE was true negative and the strain elastography was false positive for thyroid cancer. (a): The thyroid nodule in coronal plane on supersonic shear wave elastography (SSWE) (upper image) and B-mode ultrasound (lower image). The nodule is very soft on SSWE in contradiction to strain elastography (b). In the centre of the circular ROIs punctuate increased stiffness of small calcifications that are also visible on B-mode (lower image) is seen. Also artifacts of increased stiffness in the isthmus of the thyroid are visible. These artifacts are due to trachea (star arrow) and can be avoided with imaging in paracoronal plane of the nodule that does not incorporate the trachea. (b): The thyroid nodule (ROI 1-yellow) on strain elastography in sagittal plane, where the comparison with upper part of normal tissue of isthmus (ROI 2-pink) is possible. The strain color map (left) presents mostly stiff nodule-thyroid nodule score 3. The time-strain graph (right) presents that the lesion is more than 2 times stiffer than thyroid tissue.

However, imaging with SSWE, as a sensitive method of evaluation of stiffness of human tissue, the operator should be aware of physiological processes influencing the elasticity and easily apply a few rules to avoid artifacts (Figures 4, 5 and 6) (Supplementary material 1-cine loop video). Among well-known artifacts on SSWE that should be mentioned is the one that can be encountered in any region when the SSWE can be applied: the increased stiffness of the structures under externally applied pressure (Figures 4 and 5) (Supplementary material 1-cine loop video) that can be due to nonlinear elastic effects, well explained by theory [25]. Another artifact that can be encountered in thyroid SSWE is one of increased stiffness in the isthmus of the thyroid due to trachea (Figure 6). It can be avoided with imaging in paracoronal plane of the nodule that does not incorporate the trachea. However, it is important to state that these artifacts when properly interpreted do not hinder the accurate diagnosis. 

Supersonic shear wave elastography may add a new dimension to ultrasound evaluation of thyroid nodules in several ways, for example:

- improve general performance in elasticity differentiation of thyroid nodules over strain elastography due to its high reproducibility, independence of examiners skill and numeral scale of elasticity measurement in kPa;
- overcome the limitations of strain elastography:
  - nodules with liquid components or with degenerative changes;
  - small nodules (very good spatial resolution of the technique);
  - large nodules (possibility of subsequent determination of stiff regions even of large nodules, without the need of visualizing the whole nodule on one image);
  - multinodular goiter with no or scarce normal thyroid tissue as a reference;
- differentiation between soft-inspissated colloid and stiff microcalcifications;
- visualization of microcalcifications, even not visualized on B-mode imaging (may increase sensitivity and decrease specificity of thyroid cancer diagnosis);
- introduction of three-dimensional elastographic images to routine clinical practice and to national thyroid cancer databases [26], as this technique is already available and enables rapid acquisition of 3D ultrasound and elastographic data. This would devoid diagnostic process and data archiving of image selection bias attributable to 2D ultrasound examination.

Further multicenter large scale studies of thyroid nodules evaluating different elastographic methods are warranted, including (a) investigation of developmental models of diseases that link biomechanical properties (elastography findings) with genetic, cellular, biochemical, and gross pathological changes; (b) comparison of accuracy of different elastographic methods; (c) establishment of optimal diagnostic elastographic criteria; (d) establishment of limitations of different elastographic methods in relation to evaluation of thyroid pathology.

## Supplementary Material

Supplementary Material: cine loop video. An artifact in the isthmus of thyroid from tissue compression on elastogram *(*upper image) in patient with Hashimoto thyroiditis, with no real focal lesions evident on B-mode ultrasound *(*lower image) *[*Fig 5*]*. A hard stiff pseudolesion is generated during the compression with ultrasound probe and resolved during decompression. To avoid such artifacts no pressure on the probe during elasticity evaluation should be applied.

## Abbreviations

FNB: Fine-needle biopsy
SSWE: Supersonic shear wave elastography
kPa: Kilo-Pascal.

## References

[1] Tonacchera M, Pinchera A, Vitti P (2010). Assessment of nodular goiter. *Best Practice & Research*.

[2] Khati N, Adamson T, Johnson KS, Hill MC (2003). Ultrasound of the thyroid and parathyroid glands. *Ultrasound Quarterly*.

[3] Frates MC, Benson CB, Doubilet PM (2006). Prevalence and distribution of carcinoma in patients with solitary and multiple thyroid nodules on sonography. *Journal of Clinical Endocrinology and Metabolism*.

[4] Slapa R, Jakubowski W, Slowinska-Srzednicka J, Bierca J, Szopinski K How can new ultrasound techniques that enhance visualization of calcification/microcalcification-like echogenic foci influence the ultrasound evaluation of thyroid nodules?.

[5] Garra BS (2007). Imaging and estimation of tissue elasticity by ultrasound. *Ultrasound Quarterly*.

[6] Ophir J, Cespedes I, Ponnekanti H, Yazdi Y, Li X (1991). Elastography: a quantitative method for imaging the elasticity of biological tissues. *Ultrasonic Imaging*.

[7] Varghese T (2009). Quasi-static ultrasound elastography. *Ultrasound Clinics*.

[8] Parker KJ, Doyley MM, Rubens DJ (2011). Imaging the elastic properties of tissue: the 20 year perspective. *Physics in Medicine and Biology*.

[9] Vorländer C, Wolff J, Saalabian S, Lienenlüke RH, Wahl RA (2010). Real-time ultrasound elastography-a noninvasive diagnostic procedure for evaluating dominant thyroid nodules. *Langenbeck’s Archives of Surgery*.

[10] Rago T, Scutari M, Santini F (2010). Real-time elastography: useful tool for refining the presurgical diagnosis in thyroid nodules with inderterminate or nondiagnostic cytology. *The Journal of Clinical Endocrinology & Metabolism*.

[11] Lippolis PV, Tognini S, Materazzi G (2011). Is elastography actually useful in presurgical selection of thyroid nodules with indeterminate cytology?. *The Journal of Clinical Endocrinology & Metabolism*.

[12] Kagoya R, Monobe H, Tojima H (2010). Utility of elastography for differential diagnosis of benign and malignant thyroid nodules. *Otolaryngology*.

[13] Bhatia KSS, Rasalkar DP, Lee YP (2011). Cystic change in thyroid nodules: a confounding factor for real-time qualitative thyroid ultrasound elastography. *Clinical Radiology*.

[14] Slapa RZ, Jakubowski WS, Bierca J, Migda B, Slowinska-Srzednicka J Diagnostic performance of strain elastography in multinodular thyroid goiter is unsatisfactory.

[15] Park SH, Kim SJ, Kim E-K, Min JK, Eun JS, Kwak JY (2009). Interobserver agreement in assessing the sonographic and elastographic features of malignant thyroid nodules. *American Journal of Roentgenology*.

[16] Cakir B, Aydin C, Korukluoglu B Which axis should be performed for elastography scoring in thyroid nodules?.

[17] Tanter M, Bercoff J, Athanasiou A (2008). Quantitative assessment of breast lesion viscoelasticity: initial clinical results using supersonic shear imaging. *Ultrasound in Medicine and Biology*.

[18] Evans A, Whelehan P, Thomson K (2010). Quantitative shear wave ultrasound elastography: initial experience in solid breast masses. *Breast Cancer Research*.

[19] Sebag F, Vaillant-Lombard J, Berbis J (2010). Shear wave elastography: a new ultrasound imaging mode for the differential diagnosis of benign and malignant thyroid nodules. *The Journal of Clinical Endocrinology & Metabolism*.

[20] Cosgrove DO, Berg WA, Dore CJ (2012). Shear wave elastography for breast masses is highly reproducible. *European Radiology*.

[21] Berg WA, Cosgrove DO, Dore CJ (2012). Shear-wave elastography improves the specificity of brest US: the BE1 multinational study of 939 masses. *Radiology*.

[22] Rubaltelli L, Corradin S, Dorigo A (2009). Differential diagnosis of benign and malignant thyroid nodules at elastosonography. *Ultraschall in der Medizin*.

[23] Cantisani V, Ricci P, Medvedeieva M (2011). Prospective evaluation of multiparametric ultrasound and quantitative elastosonography in the differential diagnosis of benign and malignant thyroid nodules. *Insights into Imaging*.

[24] Bercoff J Shear wave elastography. http://www.supersonicimagine.fr/.

[25] Gennisson J-L, Rénier M, Catheline S (2007). Acoustoelasticity in soft solids: assessment of the nonlinear shear modulus with the acoustic radiation force. *Journal of the Acoustical Society of America*.

[26] Slapa RZ, Jakubowski WS, Slowinska-Srzednicka J, Szopinski KT (2011). Advantages and disadvantages of 3D ultrasound of thyroid nodules including thin slice volume rendering. *Thyroid Research*.

